# Training Sequence Matters: Neuromuscular Training Followed by Small-Sided Games Enhances Physical Fitness and Soccer-Specific Performance in Prepubertal Soccer Players

**DOI:** 10.3390/jfmk11030319

**Published:** 2026-08-16

**Authors:** Sofiene Bouguerra, Mohamed Amine Selmi, Lotfi Bouguerra, Yassine Negra, Roland van den Tillaar, Raouf Hammami

**Affiliations:** 1 Higher Institute of Sport and Physical Education of Kef, University Campus, University of Jendouba, Jendouba 7100, Tunisia; bouguerrasofiene949@gmail.com; 2 Tunisian Research Laboratory ‘Sports Performance Optimization’, National Center of Medicine and Science in Sports (CNMSS) (CNMSS-LR09SEP01), Tunis 1004, Tunisia; selmi.med.amin@gmail.com; 3 Higher Institute of Sport and Physical Education of Ksar-Said, University Campus, University of Manouba, Manouba 2010, Tunisia; ; yassinenegra@hotmail.fr; 4 Research Laboratory (LR23JS01) Sport Performance, Health & Society, Higher Institute of Sport and Physical Education of Ksar-Said, University of Manouba, Tunis 2010, Tunisia; lotfibouguerra@yahoo.fr; 5 Department of Sport Sciences and Physical Education, Nord University, Høgskoleveien 27, 7600 Levanger, Norway

**Keywords:** growth and maturation, facilitating effects, training order, training specificity, skills competencies

## Abstract

**Background:** Physical and technical development in youth soccer depends on the integration of neuromuscular and small-sided games (SSG) training. However, the influence of training sequence on adaptations remains unclear in prepubertal players. Therefore, this study aimed to compare the effects of performing neuromuscular training before or after SSG on physical fitness and soccer-specific performance in trained prepubertal soccer players. **Methods:** Twenty-two trained prepubertal male soccer players were randomly assigned to either neuromuscular training followed by SSG or SSG followed by neuromuscular training. Both groups completed a 6-week in-season intervention, including two weekly sessions, with each group performing 3 weeks of neuromuscular training and 3 weeks of SSG training in a different order. Physical fitness (back extensor strength, five-jump test, 10 m sprint, and Yo-Yo IR1) and soccer-specific performance (change of direction with ball and Loughborough Soccer Passing Test) were assessed before and after the intervention. **Results:** All 22 participants completed the intervention (*n* = 11 per group). Both groups demonstrated significant improvements over time in most physical fitness and soccer-specific performance variables (*p* < 0.01). However, significant group × time interactions indicated that the magnitude of improvement was consistently greater when neuromuscular training preceded small-sided games (*p* ≤ 0.041). Compared with the SSG-before-neuromuscular group, the neuromuscular-before-SSG group achieved larger improvements in back strength (+9.8% vs. +2.7%), five-jump test (+17.1% vs. +10.0%), 10 m sprint (−10.3% vs. −6.3%), Yo-Yo IR1 distance (+35.4% vs. +20.4%), change of direction with ball (−7.4% vs. −1.0%), and Loughborough Soccer Passing Test (−16.0% vs. −7.8%). **Conclusions:** Training sequence may influence the magnitude of short-term adaptations in prepubertal soccer players. Although both training orders improved performance, performing neuromuscular training before SSG resulted in greater improvements in several physical fitness and soccer-specific performance outcomes. These findings suggest that training order may represent an important factor in youth soccer training design; however, further research is required to confirm the consistency of this sequencing effect and to explore the mechanisms underlying these adaptations.

## 1. Introduction

Soccer performance depends on the integration of technical, tactical, physiological, and neuromuscular capacities [1,2]. In youth soccer, the development of physical fitness components such as sprint speed, change-of-direction (COD) ability, muscular power, balance, and aerobic fitness is particularly important because these qualities underpin successful match performance and long-term athletic development [3]. To optimize these adaptations, youth training programs commonly combine complementary approaches, particularly neuromuscular training and small-sided games (SSG). However, beyond the selection of training modalities, the order in which different stimuli are applied may represent an important factor influencing the magnitude of adaptation, especially during sensitive developmental periods. During prepuberty, the high plasticity of the neuromuscular system provides a unique opportunity for training-induced adaptations, making this stage particularly responsive to appropriately structured training interventions [4,5].

Therefore, determining whether the sequencing of neuromuscular and game-based training influences physical and soccer-specific outcomes may provide valuable information for the design of evidence-based training strategies in young soccer players.

Among the various training approaches available to youth coaches in soccer, neuromuscular training [6,7,8] and small-sided games training [9,10] have received considerable attention. Previous studies have consistently demonstrated that neuromuscular training can improve sprint performance, jumping ability, dynamic balance, movement competency, and injury resilience in youth athletes, including prepubertal soccer players [7,11]. These adaptations are primarily attributed to improvements in motor-unit recruitment, intermuscular coordination, movement efficiency, and force production capabilities, rather than morphological changes [12].

Small-sided games training, in contrast, represents a sport-specific training modality that simultaneously develops technical, tactical, and physiological capacities through modified game formats involving fewer players and reduced playing areas [13]. Research has shown that small-sided games training can improve aerobic fitness, repeated high-intensity exercise performance, technical execution, decision-making, and game-related tactical behaviors, while maintaining high levels of player motivation and enjoyment [9,10,13]. Because small-sided games closely replicate the perceptual and action demands of competitive soccer, they are widely used as an effective and ecologically valid training method during youth development [9,10,13].

Although both neuromuscular and small-sided games (SSG) training are independently effective, recent evidence suggests that the organization and sequencing of training stimuli may influence the magnitude of training adaptations. From a training periodization perspective, the order in which complementary training modalities are applied may affect how adaptations from one stimulus interact with subsequent training demands. Concepts related to training sequencing and residual adaptations suggest that previously developed physical qualities may contribute to the effectiveness of subsequent training stimuli; however, the specific mechanisms underlying these interactions remain to be established. In this regard, it has been hypothesized that neuromuscular training-induced improvements in movement quality, force production, coordination, and neuromuscular control may potentially contribute to the ability of young players to better tolerate and benefit from the higher-intensity technical and tactical demands encountered during small-sided games [14]. Conversely, initiating training with small-sided games may expose players to complex soccer-specific demands before establishing optimal neuromuscular foundations, potentially limiting subsequent adaptations.

Recent studies investigating the sequencing of different training modalities in youth soccer players have suggested that the order in which training stimuli are applied may influence performance adaptations [15,16,17]. However, current evidence has primarily focused on the sequencing of strength, plyometric, sprint, or endurance training, while the influence of sequencing between neuromuscular training and small-sided games (SSG) remains insufficiently explored. Specifically, it remains unclear whether the order of these two commonly used training modalities affects adaptations in physical fitness and soccer-specific performance in trained prepubertal soccer players.

This represents an important research gap because prepubertal players are undergoing specific neuromuscular and motor-learning development, which may influence their responses to different training approaches [6,7,8]. Therefore, the unresolved question addressed in the present study was whether performing neuromuscular training before SSG, compared with the reverse sequence, produces different short-term adaptations in physical fitness and soccer-specific performance. Although it is plausible that the order of training stimuli may influence subsequent adaptations, the mechanisms underlying such sequencing effects remain hypothetical and were not directly assessed in this study.

Accordingly, the purpose of this study was to compare the effects of two training sequences—neuromuscular training followed by SSG versus SSG followed by neuromuscular training—on physical fitness and soccer-specific performance in trained prepubertal soccer players. This investigation aimed to determine whether training order represents a relevant factor to consider when designing developmentally appropriate training strategies for young soccer players. Based on previous evidence regarding the benefits of neuromuscular training for youth athletic development [17,18,19], we hypothesized that performing neuromuscular training before SSG would result in greater improvements in physical fitness and soccer-specific performance than the reverse sequence. However, this hypothesis reflects a proposed sequencing effect, and the physiological mechanisms potentially explaining these differences were not directly examined.

## 2. Methods

### 2.1. Experimental Design and Methodology

This study employed a randomized, parallel-group, repeated-measures intervention design to examine the effects of two different training sequences involving neuromuscular training and small-sided games (SSG) on physical fitness and soccer-specific performance in prepubertal male soccer players. After baseline testing and familiarization sessions, participants were randomly allocated to one of two experimental groups: (1) neuromuscular training followed by SSG (neuromuscular-SSG group), or (2) SSG followed by neuromuscular training (SSG-neuromuscular group). Before baseline measurements, all participants completed familiarization sessions to become accustomed to the testing procedures, including the physical fitness assessments (back extensor strength, five-jump test, 10 m sprint, and Yo-Yo IR1) and soccer-specific performance tests (change-of-direction with ball and Loughborough Soccer Passing Test). These sessions were conducted under the supervision of the research team to ensure proper execution of the tests and minimize potential learning effects. Both groups completed a 6-week in-season intervention during the same competitive period (March to May 2026), integrated into their regular training schedule without modifying overall training frequency or volume. The neuromuscular-SSG group performed three weeks of neuromuscular training followed by three weeks of SSG, whereas the SSG-neuromuscular group completed the same training modalities in the opposite order. This design allowed the effects of training sequence to be compared while maintaining equivalent training exposure, duration, and volume between groups.

All participants completed identical pre- and post-intervention assessments, including anthropometric measurements, physical fitness tests (back extensor strength, five-jump test, 10 m sprint, and Yo-Yo IR1), and soccer-specific performance tests (change of direction with ball and the Loughborough Soccer Passing Test). Testing procedures, familiarization sessions, environmental conditions, and warm-up and cool-down protocols were standardized across groups.

A non-intervention control group was not included because the primary objective of this study was to compare the effects of two commonly implemented and evidence-based training sequences within an ongoing competitive-season environment. Moreover, previous studies have already demonstrated the effectiveness of neuromuscular training for improving physical fitness in prepubertal soccer players [6,7,8]. Therefore, the present design focused specifically on determining whether the order of neuromuscular training and SSG influenced the magnitude of training adaptations.

### 2.2. Participants

The sample size estimation was performed using G*Power software (version 3.1.6). Based on findings from a related study [16] that examined the sequencing effects of balance and plyometric training on 10 m sprint performance in young male soccer players (Cohen’s f = 0.61), an a priori power analysis indicated that a total sample of 8 participants would be required to achieve 80% statistical power at an alpha level of 0.05. Due to participant availability and eligibility criteria, 22 players were ultimately recruited and completed the study. Accordingly, 22 prepubertal male soccer players were recruited from a youth soccer academy center in Ariana, Tunisia (Table 1). Participants were eligible for inclusion if they were aged 10–11 years, had a minimum of two years of systematic soccer training experience, participated regularly in team training sessions and competitions, and were classified as prepubertal based on maturity offset assessment. Players were excluded if they had sustained a musculoskeletal injury within the previous six months, had any medical condition preventing participation in physical training, or were unable to complete the testing or intervention sessions. Following baseline assessments, participants were randomly allocated, using a computer-generated randomization sequence, to one of two training-sequence groups: neuromuscular training followed by small-sided games (neuromuscular-SSG group; *n* = 11) or small-sided games followed by neuromuscular training (SSG-neuromuscular group; *n* = 11). Participants’ biological maturity status was estimated based on the maturity offset method using the prediction equation of Moore et al. [20]. The participating young male prepubertal soccer players were aged 10–12 years (peak-height velocity [PHV] = −2.1 to −2.2, age at PHV = 13.1–13.5 years) (Table 1). Although the participants in the present study had previous strength and conditioning experience (1–2 years), they were still within a developmental period characterized by ongoing growth and maturation. Therefore, according to the training and performance caliber framework of McKay et al. [21], the study population can be categorized as Tier 2 (trained/developmental). For the 1–2 years preceding the study, the participating young soccer players regularly engaged in general strength and conditioning training 2–3 times per week. This training primarily consisted of traditional neuromuscular body-mass exercises (e.g., standing balance, push-ups, squats, jumps, sprint and COD). The sport-specific sessions focused on developing soccer skills such as passing, shooting, and small-sided game exercises. All players were familiar with conventional neuromuscular exercises, as this exercise type was regularly included in their previous training programs. This study was conducted following the latest version of the Declaration of Helsinki, and the protocol was approved by the local ethics committee of the National Centre of Medicine and Science of Sports, Tunis (approval code: CNMSS-LR09SEP01, approval date: 17 March 2025) before the commencement of the study. None of the participating athletes had a history of psychological and/or musculoskeletal, neurological, or orthopedic disorders six months prior to the start of the study.

### 2.3. Maturational Assessment and Anthropometry

Athletes’ body height and mass were collected using a wall-mounted stadiometer (Florham Park, NJ, USA) and an electronic scale (Baty International, West Sussex, UK), respectively. The sum of skinfolds was assessed using Harpenden’s skinfold calipers. Anthropometric testing was conducted according to Deurenberg et al. [22], who reported similar prediction errors between adults and adolescents. Thereafter, biological maturity was evaluated non-invasively using chronological age, standing and sitting height as input parameters for a regression equation to subsequently predict the maturity offset [20]. The following prediction equations were applied:

MO = −7.999994 + (0.0036124 × age × height).

The equation has previously been validated for boys and presents a standard error of estimate reported as 0.542 years [20].

### 2.4. Testing Procedure

Two weeks before the start of the study, a familiarization session was scheduled to allow the participating athletes to become acquainted with the applied tests and exercises, thereby minimizing potential learning effects during actual testing. Neuromuscular training was implemented two times per week for six weeks, with each session lasting approximately 20–25 min. Exercises were performed mostly barefoot to enhance somatosensory feedback. Programming during neuromuscular training included progression of balance, strength, jump, sprint and COD exercises over the course of the intervention. Each session comprised five exercises, 1–3 sets per exercise, and each exercise was performed for 30–40 s with a 30 s rest between sets.

Endurance training was based on small-sided games (SSG), as previously described by Enright et al. [13], using a 3 vs. 3 format without goalkeepers. The assessment of soccer-related physical fitness included a physical fitness test (muscle strength (back extensor strength) and power (five-jump test, 10 m linear sprint)), as well as sport-specific performance tests (15 m COD dribbling test without a ball) and Loughborough Soccer Passing Test. The same test sequence was applied during pre- and post-tests. Participants received standardized instructions for the technically sound performance of the physical fitness tests.

#### 2.4.1. Maximal Isometric Back Strength Performance

Maximal isometric back extensor strength was tested in kilograms using a calibrated manual dynamometer (Takei Physical Fitness Test, T.K.K. 5002 Back-A, Takei Scientific Instruments Co., Ltd., Japan) as previously described [23,24]. This measure was included as a general marker of trunk and posterior chain strength, providing insight into core stabilization and force transfer capacity during upper-body tasks [23,24]. Although push-ups primarily target the upper-body musculature, effective performance on both stable and unstable surfaces requires coordinated trunk stabilization, including engagement of the back extensors to maintain posture and optimize force production. Participants stood in an upright position with their feet shoulder-width apart and gripped the handlebar positioned across their thighs. The chain length on the force plate was adjusted so that the legs were straight, and the back was flexed at a 30° angle to position the bar at the level of the patella. Participants were then asked to straighten their back (i.e., stand upright) without bending their knees and to lift the chain, with the pulling force applied to the handle. The athletes were kindly asked to pull as forcefully as possible. The participants completed three trials, and the best trial was used for further analysis. A thirty-second rest interval was provided between trials. Previously, test–retest reliability conducted with young athletes reached an ICC of 0.98 and a SEM of 1.18% [23].

#### 2.4.2. Sprint Performance

The 10 m sprint distance was selected to evaluate acceleration, which is a critical component of match-play performance in youth soccer [25]. The 10 m sprints capture short-distance acceleration, which is particularly relevant in small-area play and transitions. Hence, the selected distance has been validated and widely used in similar populations to record speed development and training adaptations [26]. Linear sprint times were recorded using electronic photocell timing gates (Brower Timing Systems, Salt Lake City, UT, USA) with an accuracy of 0.001 s. Gates were positioned at the start line, as well as at the 10 m marks, each placed approximately 0.4 m above the ground to align with the athletes’ torso height and reduce early triggering by limb movement. Participants began each sprint from a static, two-point standing position, with their front foot placed 0.5 m behind the first timing gate to ensure the start was based on movement initiation, not a false trigger. Players were instructed to sprint maximally through the final gate. The best time from three valid trials was used for analysis, with at least 2 min of passive recovery between efforts. Test–retest reliability was excellent, with ICC = 0.91 and SEM = 0.15% [15].

#### 2.4.3. Horizontal Jump Performance

The five-jump test was used as a proxy to estimate muscle power, following the guidelines of Chamari et al. [27]. Players started the test in a standing position with both feet flat on the ground and performed five alternating left and right leg bounds, aiming to cover the maximum possible horizontal distance. As the dependent variable, the horizontal jump distance was tested to the nearest centimeter using a tape measure. This test has previously shown high test–retest reliability, with an ICC of 0.91 for youth soccer players [28].

#### 2.4.4. Yo-Yo Intermittent Recovery Test Level 1 (Yo-Yo IR1)

Intermittent endurance capacity was assessed using the Yo-Yo Intermittent Recovery Test Level 1 (Yo-Yo IR1), a valid and reliable field-based test widely used in youth soccer players to evaluate the ability to repeatedly perform intense exercise interspersed with brief recovery periods [29]. The test was performed on a flat outdoor surface according to the standardized protocol described by Bangsbo et al. [29]. Participants performed repeated 2 × 20 m shuttle runs at progressively increasing speeds dictated by audio signals emitted from a prerecorded soundtrack. Between each shuttle bout, players completed a 10 s active recovery period consisting of 2 × 5 m jogging around a cone positioned behind the starting line. Running speed increased progressively throughout the test until volitional exhaustion or until the participant failed on two consecutive occasions to reach the designated line in time with the audio signal. Standardized verbal encouragement was provided throughout the test to ensure maximal effort. The total distance covered (m) before termination was recorded as the performance score and was subsequently used to estimate maximal aerobic speed and maximal oxygen uptake (VO_2_max) according to previously established equations [29]. The Yo-Yo IR1 has demonstrated excellent reliability in youth soccer players, with reported ICC of 0.94 and an SEM of 3% [30,31], supporting its sensitivity for detecting training-induced changes in intermittent endurance performance.

#### 2.4.5. Sport Specific Performance Tests

**Figure 1:**
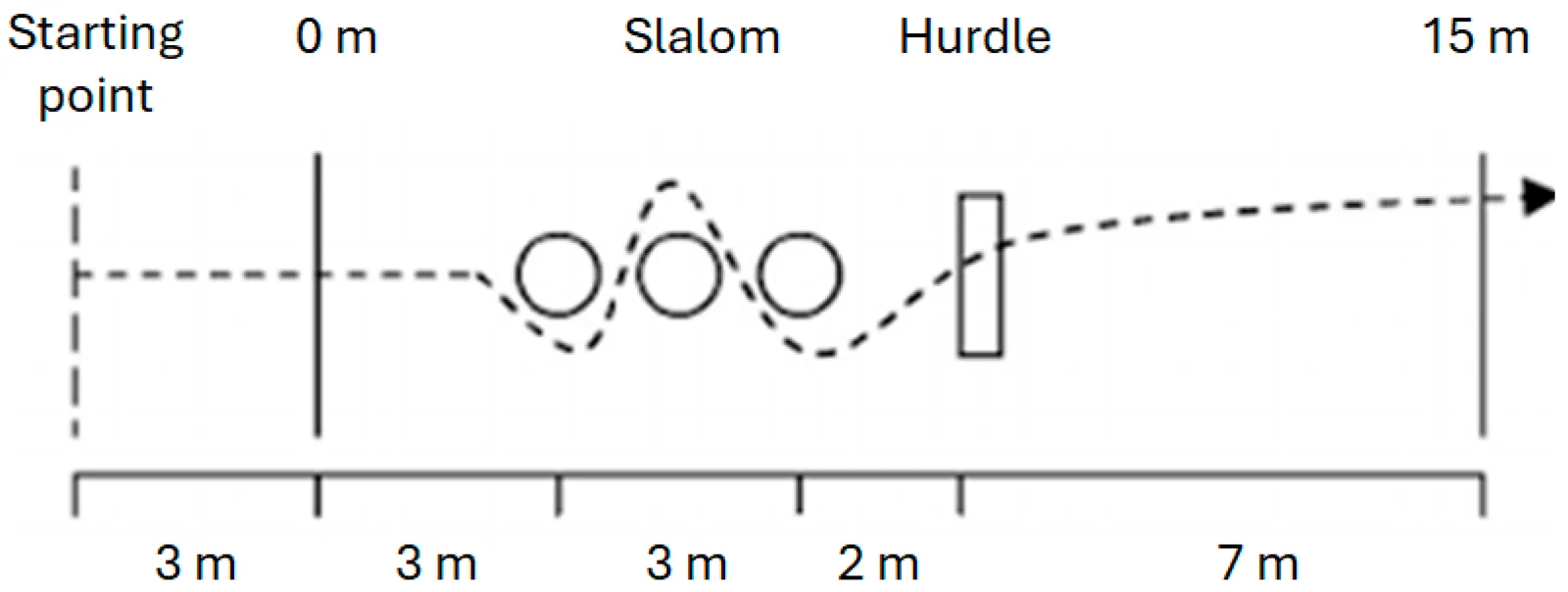
Schematic representation of the change-of-direction test with ball.

The sport-specific performance tests were a change-of-direction (COD) ability test with ball and the Loughborough Soccer Passing Test. The COD with ball test was designed to evaluate players’ ability to rapidly accelerate, decelerate, and change direction while simultaneously maintaining precise ball control, thereby providing a more sport-specific assessment of agility and technical performance under time pressure. The 15 m COD test required participants to run from 3 m behind the start line, complete a 3 m straight sprint, navigate a 3 m slalom (three poles, 1.5 m apart), clear a 0.5 m hurdle placed 2 m beyond the final pole, and sprint 7 m to the finish gates (Figure 1) [32]. During the test, participants were required to complete the course while dribbling a standard football (circumference: 68–70 cm [27–28 cm in]; mass: 410–450 g), continuously controlling the ball using their preferred foot. Players were instructed to perform each trial at maximal effort, emphasizing both speed of movement and accuracy of ball handling, with the aim of minimizing total completion time without losing control of the ball or committing technical errors that could affect performance. Each participant performed two trials, with a standardized 3 min passive recovery period between attempts to reduce fatigue-related effects and ensure optimal performance in subsequent trials. The best (fastest) performance across the two trials was retained for statistical analysis to represent each player’s COD performance with ball. The test has demonstrated excellent measurement properties in previous laboratory-based validations, showing high test–retest reliability (ICC = 0.90) and low measurement error (SEM = 0.46%), supporting its use as a stable and sensitive indicator of soccer-specific agility and technical performance [15].

**Figure 2:**
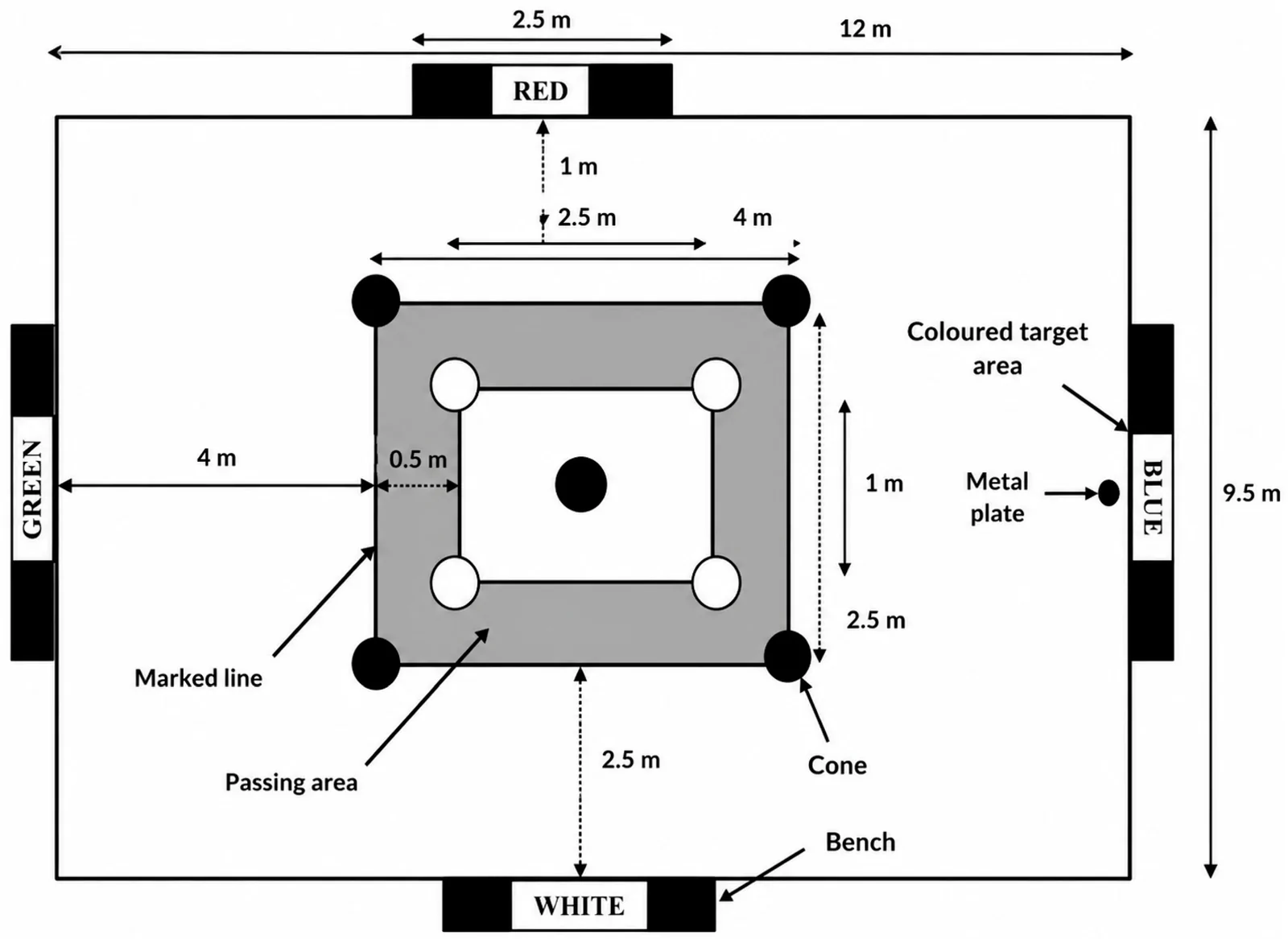
Schematic representation of the Loughborough Soccer Passing Test protocol and scoring system.

Passing performance was assessed using the Loughborough Soccer Passing Test [33], a standardized and reliable field-based test designed to evaluate soccer-specific passing accuracy and technical performance under time pressure (Figure 2). The test was conducted in a marked area containing multiple numbered target panels positioned around the player at different angles and distances. Participants started from a central position and were required to complete a predefined sequence of passes to the target panels as quickly and accurately as possible, using the appropriate foot and following the prescribed order. After each pass, the ball was controlled and returned to the central area before continuing to the next target according to the standardized protocol.

Performance was expressed as a composite score combining completion time and penalty time. Penalties were added for execution errors such as inaccurate passes, incorrect target selection, or failure to strike the target correctly. The final score was calculated as: Final score = completion time + penalty time, with lower scores indicating better performance. All participants completed a familiarization session prior to testing to minimize learning effects and improve measurement consistency. The Loughborough Soccer Passing Test has demonstrated excellent reliability, with reported ICC ranging from 0.90 to 0.95 and an SEM of approximately 2–5%, supporting its sensitivity for detecting changes in soccer passing performance [33].

### 2.5. Training Intervention

Both training programs lasted six weeks and were integrated into the players’ in-season soccer training schedule from March to May 2026. Before the intervention, all participants followed a typical in-season routine consisting of five weekly soccer training sessions (Tuesday to Saturday), with Sunday reserved for competition and Monday for recovery. Players had no previous experience with general soccer conditioning activities, including basic balance, strength, jumping, sprinting, and change-of-direction exercises and a structured neuromuscular training program or a systematic small-sided games (SSG) intervention (1–2 years). Therefore, both interventions represented novel structured training stimuli.

Each 90 min training session began with a standardized 15 min dynamic warm-up, including dynamic stretching, submaximal running, acceleration and deceleration drills, and jump-landing tasks. On Tuesdays and Thursdays, a 30 min training block consisting of either neuromuscular training or SSG replaced an equivalent portion of the regular soccer training content. Following this block, players completed 40 min of soccer-specific training, including 20 min of technical-tactical exercises and 20 min of SSG activities with or without goals. On Wednesdays, Fridays, and Saturdays, players performed 70 min of soccer-specific training after the warm-up, consisting of 35 min of technical-tactical drills and 35 min of SSG-based activities. All sessions concluded with a standardized 5 min cool-down period (Table 2).

The training sequence differed between groups. The neuromuscular-SSG group completed three weeks of neuromuscular training followed by three weeks of SSG, whereas the SSG-neuromuscular group performed the same training modalities in the reverse order. Training exposure was matched between groups in terms of session duration, weekly frequency, and total intervention duration. However, because neuromuscular training and SSG represent distinct training modalities, intensity was not directly equated between conditions. Instead, both groups followed the same overall training schedule and were exposed to equivalent amounts of structured training time. Internal and external training loads during SSG sessions were not objectively monitored, which should be considered a limitation of the study. All sessions were conducted on the same soccer pitch under similar environmental and contextual conditions.

The neuromuscular training program consisted of five exercises targeting balance, strength-power development, linear sprinting, change-of-direction speed, and agility. Players performed three sets of 5–10 repetitions per exercise, with 60–120 s of passive recovery between sets and exercises [6]. Progressive overload was implemented by adjusting exercise difficulty and perceived exertion every two weeks using the 0–10 OMNI scale. Target RPE values progressed from 3 to 4 during weeks 1–2, to 5–6 during weeks 3–4, and 7–8 during weeks 5–6. All neuromuscular training sessions were performed on the soccer pitch to maintain ecological validity and promote transfer to soccer-specific movements (Table 3).

The SSG intervention consisted of 3 vs. 3 games performed on a 20 × 20 m playing area. Sessions included repeated game bouts with standardized work-to-rest intervals, coach encouragement to maintain high-intensity efforts, and continuous ball availability. The number and duration of bouts, recovery periods, and task constraints were progressively adjusted across the intervention period to increase the training stimulus while maintaining the same total training duration as the neuromuscular training condition.

The SSG program was designed as a soccer-specific conditioning method aimed at simultaneously developing physical fitness, technical execution, and decision-making under high physiological demand [13]. All SSG sessions were conducted on a natural grass pitch using a standardized playing area of 20 m × 20 m, consistent with previous research demonstrating that such dimensions elicit high-intensity physiological responses [13].

### 2.6. Statistics

All data analyses were performed using JASP v. 0.95.1 (University of Amsterdam, Amsterdam, The Netherlands). Data are presented as means and 95% confidence intervals (95% CI). The normality assumption was tested and confirmed using the Shapiro–Wilk test. To examine the effects of the interventions on the dependent variables, a 2 (group: neuromuscular-SSG vs. SSG-neuromuscular training) × 2 (time: pre vs. post) repeated-measures ANOVA was performed for each parameter. Given the multiple outcomes analyzed, Holm–Bonferroni correction was applied to control for multiple comparisons. To determine the magnitude of the training effects within and between groups, Cohen’s d effect sizes (ES) were calculated. Within-group ES were calculated using the standardized mean difference between pre- and post-intervention values, based on the pooled standard deviation. For the group × time interaction effects, between-group ES were calculated using the standardized difference in change scores between groups (i.e., difference-in-differences approach) divided by the pooled baseline standard deviation. According to ES values, the effects were classified as trivial (<0.2), small (0.2–0.6), moderate (0.6–1.2), large (1.2–2.0), very large (2.0–4.0), and extremely large (>4.0). To control for multiple comparisons across dependent variables, *p*-values associated with the group × time interaction effects were adjusted using the Holm–Bonferroni correction. The alpha level of significance was set at *p* < 0.05.

## 3. Results

All participants completed the intervention as allocated, and no training- or testing-related injuries were reported. All variables met the assumptions of normality. Baseline characteristics, maturity offset, and performance variables were comparable between groups, with no meaningful differences observed (Table 1).

### 3.1. Physical Fitness

**Figure 3:**
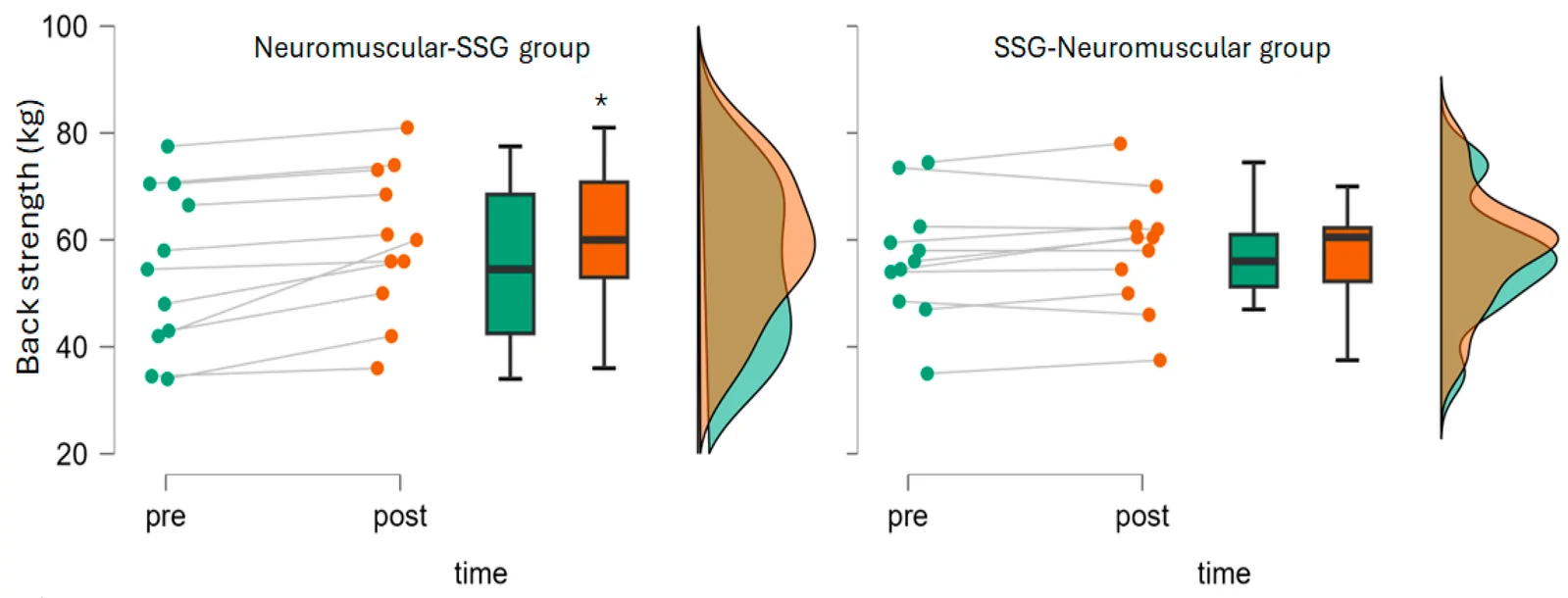
Mean (95% CI) maximal individual isometric back strength per individual and group at pre- and post-test. * indicates a significant difference from pre- to post-test.

A significant main effect of time was observed for back strength (F = 15.80, *p* < 0.001, d = 1.78), with a significant group × time interaction (F = 4.96, adjusted *p* = 0.038, d = 1.00). Post hoc analyses showed a significant increase in back strength in the neuromuscular-SSG group (*p* = 0.005, d = 0.38, Δ = +9.78%), whereas no significant change was observed in the SSG-neuromuscular group (*p* = 0.123, d = 0.14, Δ = +2.65%) (Figure 3).

**Figure 4:**
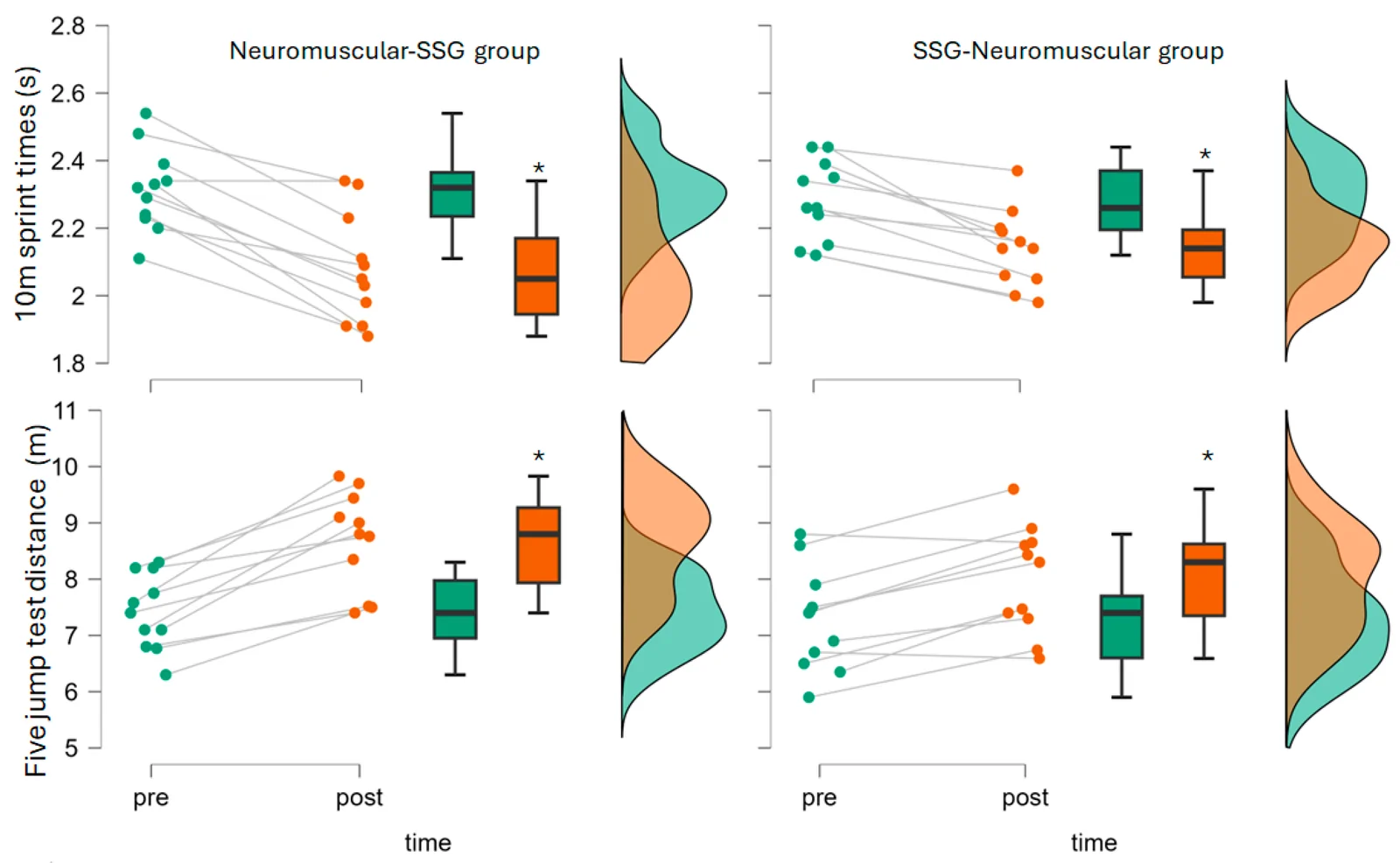
Mean (95% CI) maximal five jump and 10 m sprint performances per individual and group at pre- and post-test. * indicates a significant difference from pre- to post-test.

Significant main effects of time (F ≥ 78.75, *p* < 0.001, d ≥ 3.96) and group × time interactions (F ≥ 4.76, adjusted *p* ≤ 0.041, d ≥ 0.97) were also observed for five-jump performance and 10 m sprint time. Post hoc analyses demonstrated significant improvements in both groups: the neuromuscular-SSG group improved five-jump performance (d = 1.70, Δ = +17.1%) and 10 m sprint time (d = 1.78, Δ = −10.3%), while the SSG-neuromuscular group also improved five-jump performance (d = 0.81, Δ = +10.0%) and 10 m sprint time (d = 1.28, Δ = −6.3%). However, the magnitude of improvement was significantly greater in the neuromuscular-SSG group (Figure 4).

### 3.2. Yo-Yo IR1 Performance

**Figure 5:**
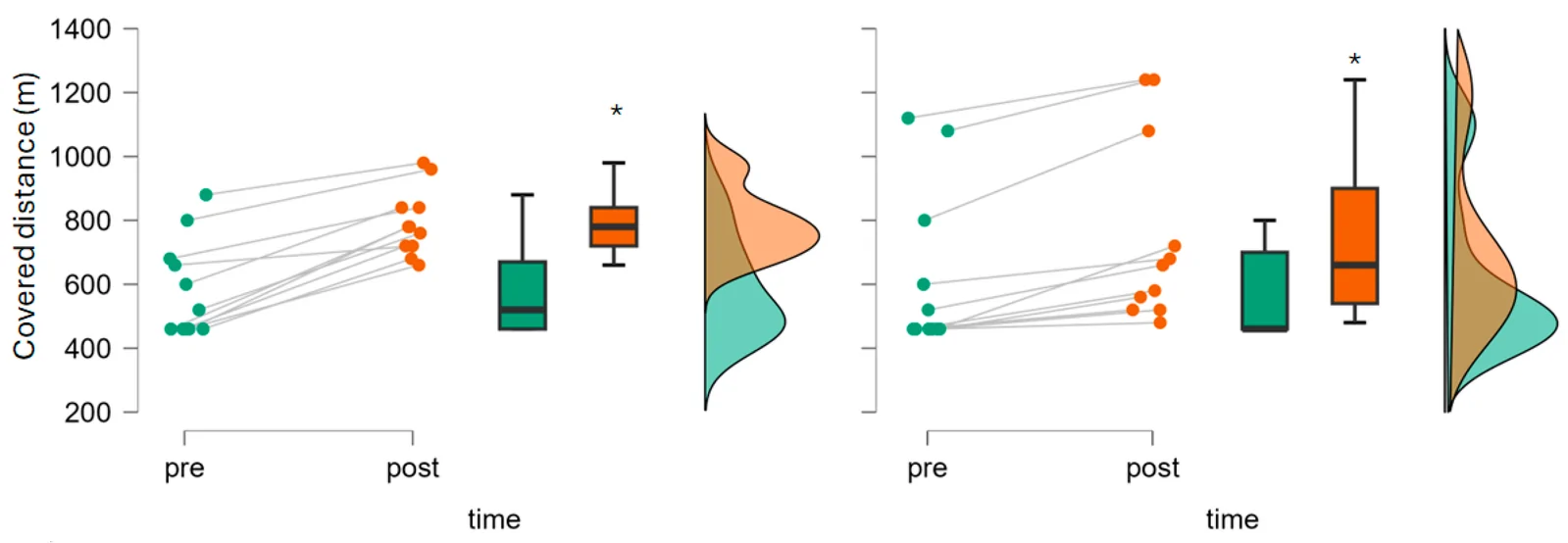
Mean (95% CI) maximal covered distance during Yo-Yo IR 1 test per individual and group at pre- and post-test. * indicates a significant difference from pre- to post-test.

A significant main effect of time was observed for total distance covered during the Yo-Yo IR1 test (F = 91.85, *p* < 0.001, d = 4.28), together with a significant group × time interaction (F = 5.25, adjusted *p* = 0.033, d = 1.02). Post hoc analyses revealed significant increases in total distance covered in both groups (neuromuscular-SSG: d = 1.67, Δ = +35.4%; SSG-neuromuscular: d = 0.49, Δ = +20.4%). However, the magnitude of improvement was significantly greater in the neuromuscular-SSG group (Figure 5).

Similar effects were observed for estimated maximal aerobic speed and VO_2_max, with significant main effects of time (F ≥ 92.17, *p* < 0.001, d ≥ 4.30) and group × time interactions (F = 5.26, adjusted *p* = 0.033, d = 1.02). Post hoc analyses showed significant improvements in maximal aerobic speed and VO_2_max in both groups, with greater improvements observed in the neuromuscular-SSG group (Table 4).

### 3.3. Soccer-Specific Performance

**Figure 6:**
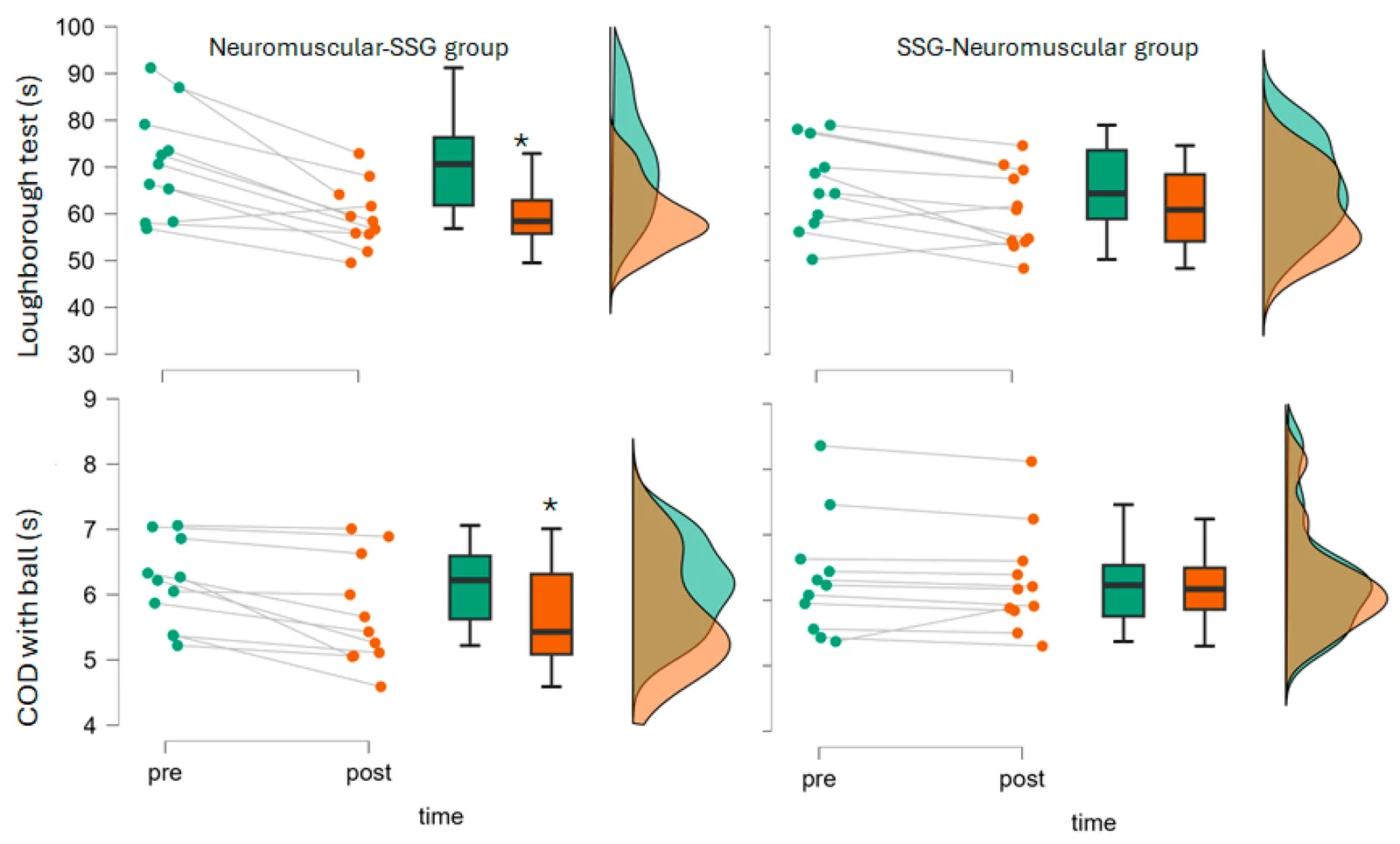
Mean (95% CI) Loughborough Soccer Passing Test and COD with ball scores per individual and group at pre- and post-test. * indicates a significant difference from pre- to post-test.

Significant main effects of time were observed for the Loughborough Soccer Passing Test and 15 m COD with ball performance (F ≥ 14.45, *p* ≤ 0.001, d ≥ 1.70). Significant group × time interactions were also detected for both outcomes (F ≥ 4.65, adjusted *p* ≤ 0.043, d ≥ 0.97). Post hoc analyses showed significant reductions in completion time in the neuromuscular-SSG group (Loughborough Soccer Passing Test: d = 1.22, Δ = −16.0%; COD with ball: d = 0.53, Δ = −7.4%). In contrast, the SSG-neuromuscular group did not demonstrate significant changes (Loughborough Soccer Passing Test: *p* = 0.093, d = 0.60, Δ = −7.84%; COD with ball: *p* = 1.00, d = 0.15, Δ = −1.0%). Consequently, the magnitude of pre- to post-intervention changes was significantly greater in the neuromuscular-SSG group (Figure 6).

## 4. Discussion

The present study is the first to examine the sequencing effects of neuromuscular and small-sided games training in trained prepubertal soccer players. Although both sequences improved performance, neuromuscular followed by SSG training consistently produced greater gains in strength, power, sprint, aerobic fitness, COD with the ball, and passing performance. These findings extend previous research by showing that training order is a key determinant of adaptation, rather than adaptations being simply additive across methods [15,16,17]. Overall, the results suggest a hierarchical relationship in which neuromuscular development enhances the effectiveness of subsequent game-based training, supporting the principles of training specificity [34] and post-activation performance enhancement [35] and indicating that SSG may be more effective when preceded by a neuromuscular foundation.

The greater improvement in back strength following the neuromuscular-SSG training sequence aligns with previous studies showing that neuromuscular training can enhance strength-related outcomes in prepubertal soccer players [6,36]. These adaptations have been proposed to involve neural factors, such as improved motor-unit recruitment and intermuscular coordination, given the limited hypertrophic potential before puberty [37]. However, these mechanisms were not directly assessed in the present study and should therefore be considered potential explanatory hypotheses. In contrast, SSG-based interventions generally demonstrate limited effects on maximal strength [38], which may help explain the smaller strength improvements observed in the SSG-neuromuscular group. The present findings suggest that the order of training modalities may influence short-term strength adaptations; however, the mechanisms underlying this potential sequencing effect remain unclear.

Both groups improved five-jump test performance, but the magnitude was significantly greater in the neuromuscular-SSG group. Both groups improved five-jump performance; however, the magnitude of improvement was greater in the neuromuscular-SSG group. These findings may indicate that SSG can contribute to the expression and transfer of physical qualities developed through complementary training modalities rather than acting as the sole stimulus for power development [39]. Previous research suggests that neuromuscular training may influence factors related to eccentric control and stretch-shortening cycle function [40], while subsequent SSG exposure may provide an environment for applying these qualities under sport-specific perceptual and decision-making constraints [41,42]. Nevertheless, these interpretations remain hypothetical because neuromuscular characteristics were not directly measured in the present study.

The greater sprint improvements observed in the neuromuscular-SSG group are consistent with previous evidence demonstrating positive effects of neuromuscular training on short-distance sprint performance in youth soccer players [43]. Although SSG can improve sprint ability, its effects may vary depending on task constraints, player numbers, and the intensity achieved during training [38,39]. The present results suggest that the sequence in which training stimuli are applied may influence sprint adaptations; however, potential explanations involving changes in force production, sprint mechanics, or movement efficiency were not directly evaluated and should be considered possible mechanisms requiring further investigation [44].

Similarly, both groups improved aerobic fitness, with greater gains observed in the neuromuscular-SSG group. While SSG is an established method for improving aerobic capacity, recent evidence suggests that aerobic adaptations may also be influenced by factors such as movement efficiency and the ability to repeatedly perform high-intensity actions during games [41,45]. The current findings may indicate that the order of neuromuscular and SSG training influences the magnitude of aerobic adaptations; however, further studies incorporating direct physiological and biomechanical assessments are required to clarify the mechanisms involved.

A notable finding was that improvements in soccer-specific performance, including change-of-direction ability with the ball and passing ability, were greater following the neuromuscular-SSG sequence. Change-of-direction performance is influenced by factors such as eccentric strength, braking capacity, and postural control [46], whereas technical performance under pressure may depend partly on balance, coordination, and stability [47]. Therefore, the observed improvements may reflect interactions between physical and technical adaptations, although the specific contributions of these factors cannot be confirmed because they were not directly assessed.

Although the observed improvements were substantial, particularly for Yo-Yo IR1 performance (+35.4% in the neuromuscular-SSG group), they should be interpreted cautiously. The magnitude of change following a six-week intervention may partly reflect factors such as learning, familiarization, and increased test experience, despite the inclusion of familiarization sessions before baseline testing. Future studies should consider repeated baseline assessments, extended familiarization procedures, and longer follow-up periods to better distinguish training-related adaptations from potential measurement effects. Finally, the interpretation of the sequencing effect should consider that post-intervention testing was conducted immediately after different final three-week training blocks. Therefore, the observed differences between groups may partly reflect recency or order effects associated with the last training modality performed rather than solely a beneficial carry-over effect of one modality on the subsequent one. Future studies using longer interventions, additional assessment points, or alternative sequencing designs are needed to further clarify the independent contribution of training order.

Despite these findings, several limitations should be acknowledged. First, although the sample size was determined through an a priori power analysis, the final sample remained relatively small (*n* = 22), which may have reduced statistical precision and limited the generalizability of the findings. Future studies with larger samples and the inclusion of repeated anthropometric assessments throughout the intervention are warranted to better account for growth-related changes and further minimize potential confounding factors, particularly in the absence of a non-intervention control group. Second, the intervention lasted only six weeks, allowing the detection of short-term adaptations but limiting conclusions regarding long-term effects. Future studies should incorporate longer interventions and follow-up assessments to determine whether sequencing effects are maintained over time. Third, the sample consisted exclusively of trained male prepubertal soccer players, whose physiological, neuromuscular, and maturational characteristics may influence training responses. Therefore, the findings should not be generalized directly to female athletes, adult players, recreational populations, or athletes from different competitive levels. Fourth, despite the standardization of the protocol, uncontrolled factors such as dietary habits, sleep, additional physical activity, and individual maturation differences may have influenced individual responses. Fifth, the absence of a non-intervention control group limits the ability to completely distinguish adaptations related to the training interventions from changes associated with normal growth and maturation. Therefore, the findings should be interpreted primarily as differences in adaptation between training sequences rather than as definitive evidence of absolute training effects. Finally, no post-intervention follow-up was conducted, preventing evaluation of the long-term retention and practical relevance of the observed adaptations.

## 5. Conclusions

The present study suggests that the sequencing of neuromuscular and small-sided games (SSG) training may influence short-term adaptations in trained prepubertal soccer players. Although both training sequences resulted in improvements in physical fitness and soccer-specific performance, performing neuromuscular training before SSG was associated with greater improvements in maximal strength, lower-limb power, sprint performance, aerobic capacity, change-of-direction ability with the ball, and technical performance. These findings provide preliminary evidence that the order in which training stimuli are applied may represent a factor influencing short-term adaptations during youth soccer development.

From a practical point of view, the current results suggest that coaches may consider the potential value of placing neuromuscular training before SSG within youth training programs. However, this should be viewed as a preliminary strategy rather than a definitive recommendation, as the present study examined only one specific sequencing approach over a six-week period. Given the limited intervention duration, relatively small sample size, single-center design, and inclusion of only trained prepubertal male players, further research involving larger and more diverse populations, longer interventions, and different competitive contexts is required before broader practical applications can be established.

Finally, this study provides initial evidence that training sequence may be a relevant consideration when designing developmentally appropriate training programs for prepubertal soccer players. Future investigations should examine the consistency of these findings, the mechanisms underlying potential sequencing effects, and their long-term implications across different populations and training environments.

## Figures and Tables

**Table 1:** Anthropometric characteristics of the included participants.

Group	Neuromuscular-SSG (*n* = 11)	SSG-Neuromuscular (*n* = 11)
Age (years)	11.4 ± 0.7	10.9 ± 0.9
Body height (m)	1.43 ± 0.09	1.47 ± 0.06
Body mass (kg)	40.5 ± 10.9	39.9 ± 7.8
Maturity offset (years)	−2.1 ± 0.6	−2.2 ± 0.7
Age PHV (years)	13.5 ± 0.4	13.1 ± 0.3

**Table 2:** Overview of training program design and weekly organization during the 6-week intervention.

Component	Description
Intervention duration	6 weeks (in-season period: March–May 2026)
Participants’ baseline routine	5 soccer sessions/week (Tuesday–Saturday), match on Sunday, recovery on Monday
Training integration	neuromuscular training or SSG integrated into regular soccer training
Session duration	90 min per session
Warm-up (all sessions)	15 min standardized dynamic warm-up (dynamic stretching, submaximal running, acceleration/deceleration, jump–landing tasks)
Main modification (Tuesday & Thursday)	30 min neuromuscular or SSG training replacing part of regular soccer training
Post-block training (Tuesday & Thursday)	40 min soccer training (20 min technical–tactical + 20 min small-sided games)
Wednesday, Friday, Saturday	70 min soccer training (35 min technical–tactical + 35 min small-sided games)
Cool-down (all sessions)	5 min standardized recovery
Neuromuscular-SSG group	Weeks 1–3: neuromuscular training → Weeks 4–6: small-sided games training
SSG-Neuromuscular group	Weeks 1–3: small-sided games training → Weeks 4–6: neuromuscular training
Training load control	Volume and intensity matched between groups
Environment	All sessions performed on natural grass soccer pitch under similar conditions
Novelty of stimulus	No prior exposure to structured neuromuscular training or SSG

**Table 3:** Neuromuscular training program design, exercise prescription, and examples of training content.

Component	Description
Training focus	Balance, strength–power, sprinting, change-of-direction speed, and agility development
Number of exercises	5 exercises per session
Exercise structure	3 sets × 5–10 repetitions per exercise
Rest intervals	60–120 s between sets and exercises
Training surface	Soccer pitch (to maintain ecological validity and sport-specific context)
Progressive overload method	Adjustment of exercise difficulty and perceived exertion using the OMNI RPE scale (0–10)
Weeks 1–2	RPE 3–4 (low–moderate intensity), emphasis on technical execution and movement control
Weeks 3–4	RPE 5–6 (moderate intensity), progression of exercise complexity and intensity
Weeks 5–6	RPE 7–8 (high intensity), progression toward maximal execution and sport-specific demands
Exercise categories and examples	Balance: single-leg stance, single-leg balance with perturbations; strength–power: bodyweight squats, jump squats, bounding; plyometric training: countermovement jumps, lateral jumps, hurdle jumps; sprint training: short acceleration drills (5–10 m); change-of-direction and agility: planned and reactive cutting drills, shuttle runs
Session goal	Improve neuromuscular control, explosive performance, movement coordination, and the ability to efficiently perform soccer-specific movements

**Table 4:** Estimated maximum aerobic speed and oxygen uptake per group at pre- and post-test.

Group	Neuromuscular-SSG		SSG-Neuromuscular	
Test	Pre	Post	Pre	Post
Maximum aerobic speed (km/h)	11.80 ± 0.37	12.30 ± 0.25 *	11.90 ± 0.62	12.21 ± 0.70 *
Maximal oxygen uptake (ml/kg/min)	41.32 ± 1.28	43.06 ± 0.88 *	41.65 ± 2.16	42.72 ± 2.45 *

* indicates a significant difference from pre- to post-test.

## Data Availability

The raw data supporting the conclusions of this article will be made available by the authors on request.
